# Frailty and Geriatric Medicine During the Pandemic

**DOI:** 10.3389/fmed.2021.673814

**Published:** 2021-06-07

**Authors:** Silvia Crosignani, Jacopo Fantinati, Matteo Cesari

**Affiliations:** ^1^Fellowship in Geriatrics and Gerontology, University of Milan, Milan, Italy; ^2^Department of Clinical Sciences and Community Health, University of Milan, Milan, Italy; ^3^Istituti di Ricovero e Cura a Carattere Scientifico Istituti Clinici Scientifici Maugeri, University of Milan, Milan, Italy

**Keywords:** aging, geriatrics, prevention, SARS-CoV2, ageism

## Abstract

The term *frailty* in the era of coronavirus disease 2019 (COVID-19) has a manifold implication. The vast majority of the countries worldwide being hit by the pandemic have shown the frailty of their health and social care systems. Although the surprise factor could somehow justify the unpreparedness experienced during the first wave, the second wave still led to significant difficulties almost everywhere. Looking at Italy's situation, it is evident how the stress test applied by COVID-19 on the system has threatened its stability, getting it closer to collapsing many times. It is true that Italy, in particular the Northern regions, has been the epicenter of COVID-19 in Europe in a time when information about the severe acute respiratory syndrome coronavirus 2 was still lacking and confusing. Nevertheless, what happened has demonstrated significant issues in the structure, priorities, and organization of the system. It has exemplified the obsolete approach adopted in clinical practice, particularly when applied to frail older persons. The COVID-19 pandemic has made emerging the need for a substantial reshaping of our healthcare system. The hospital-centered model has dramatically failed. To adequately face the new challenges brought by the increasing complexity of our aging society, it is critical to move the barycenter of action toward the community/primary care, promoting the integration of services and centralization of clinical/administrative data. It is vital to train healthcare professionals in the identification and basic principles of geriatric conditions, clarifying the role that geriatricians play. In the present article, some cornerstone concepts of geriatric medicine (i.e., definition of geriatrics, multidisciplinarity, integrated care, and development of clinical databases for filling the evidence-based medicine gaps) are presented, explaining the challenges they have faced during the COVID-19 pandemic and possible solutions for implementing improvements in the future.

## Introduction

Many healthcare systems present significant and, unfortunately, chronic difficulties with the proper management of geriatric patients. The stress applied by the pandemic to the systems has sensibly enhanced such problems and let emerge all the inadequacy of standard clinical practices. In particular, the severe acute respiratory syndrome coronavirus 2 (SARS-CoV-2) diffusion has required the worldwide implementation of immediate countermeasures to reallocate care resources following priorities that have frequently gone into conflict with the older persons' values and needs. Geriatricians have thus found themselves at operating in scenarios that were often neglecting or overlooking the basic principles of geriatric medicine. In this article, we present a brief overview describing some of the significant issues that geriatricians have historically been fighting and that have dramatically affected the clinical routine during the pandemic. As it is well-established that making clinical decisions on the only basis of the patient's age is highly arguable under multiple perspectives, the purpose of our article is to stimulate discussions for avoiding similar situations that might occur again in the future.

## Ageism

Since the very first phases of the pandemic, it seemed that people got rapidly accustomed to daily reports of hundreds (if not thousands) of casualties, superficially accepting the dramatic epidemiological data as part of “new” normality. Of course, the parallel economic crisis fed by the pandemic may have substantially contributed to diverging the focus elsewhere, that is, toward each individual's priorities. At the same time, the risk profile of persons dying of coronavirus disease 2019 (COVID-19) characterized by old age (1), combined with (1) the preexistent individualist societal model and (2) the modified priorities brought by these difficult times, has highlighted the existence prejudices (2). COVID-19 has indeed become quite soon “a problem of older persons” in the collective perspective. And this is despite the evidence that not only older people die of COVID-19, but also the case-fatality ratios also indicate an unexplained all-cause excess of deaths in young and adult individuals during these times (3). As a matter of fact, older persons with frailty are the most severely affected as evidenced by in-hospital death rates (4).

Recently, the Società Italiana di Anestesia, Analgesia, Rianimazione e Terapia Intensiva (Italian Society of Anesthesia, Analgesia, and Intensive Care) released an update of a document published during the first wave presenting clinical ethics recommendations for the allocation of treatment in exceptionally resource-limited situations (5). Despite its wide application in Italy during the early months of the pandemic, the original document (6) had been criticized for providing directions too driven by the age criterion in allocating resources. In the second version published in January 2021 (5), the role of chronological age in decisional algorithms is deflated, giving higher priority to assessing comorbidities and clinical complexity. The new version may represent a step forward in the proper assessment of the aging individual. For example, it will be important to refine the instruments aimed at assessing the individual's health status, avoiding to inflate the weight of chronological age included in them. In this context, it would also be important to clearly explain how to use certain tools (e.g., the Clinical Frailty Scale) for avoiding that they are inappropriately adopted to legitimate subjective evaluations.

As recently published by the World Health Organization, “Governments, international agencies, and health systems have an obligation to ensure, to the best of their ability, adequate provision of healthcare for all.” Even during a pandemic, difficult choices must meet ethical criteria (7). The COVID-19 pandemic has exposed a deep and older issue (i.e., ageism) and has amplified these harmful attitudes. At the same time, this has stimulated further and fruitful debates on the issue (8). It is critical to develop new models of care based on the individual's functions and reserves, avoiding arguable surrogates as the birth date to categorize and make clinical decisions. This advancement will only be possible when society as a whole will be able to differently consider the aging process, not as something to avoid (an evident failure), but a natural phenomenon potentially full of opportunities.

## Integration of Care Within the Hospital Setting

During the first wave, an unprecedented, unexpected, and overwhelming number of persons with respiratory symptoms arrived at the emergency departments. The emergent situation of COVID-19 has frequently led to a needed reorganization of the clinical units, merging different clinical specialties into macroareas of medicine. In this new framework, geriatricians have contributed to the management of patients with COVID-19 together with colleagues following the traditional non-geriatric standards.

Despite well-established scientific evidence explaining the importance of implementing geriatric models in the acute care setting (9), geriatricians' role has often remained marginal (at best). It has been quite immediately evident to geriatricians how many geriatric syndromes are completely neglected outside of their world. In particular, it has become clearly evident to geriatricians how many cases of geriatric syndromes (e.g., delirium, malnutrition, hypomobility, communication impairment, social isolation) are not recognized in the usual routine. The lack of awareness and mistreatment of these conditions outside of the geriatric units was already known, at least for those geriatricians frequently called in consultation. However, working in the “outside world” with the dynamics and methodologies planned by non-geriatricians has been extremely frustrating.

The pandemic has shown how the current system is not integrated but is designed to work as a “disease factory.” The different specialists operate per silo, and even when sharing the common spaces of the COVID-19 clinical macroareas, the exchanges on the patient were limited at passing rather than discussing information.

It frequently seems as clinicians work with the assumption that all treatments and interventions are beneficial to everyone. There is still not an adequate perception of the collateral damages that our practices might generate to the frailest patients in need of personalized care. It might be sufficient to pay more attention to concepts like the “iatrogenic disability” proposed by the International Association of Gerontology and Geriatrics (10) to realize how harmful some routine practices might become following the current “one-fits-all” model of care.

It is critical to developing a multidisciplinary environment where all the health professionals act at the same level, in respect of their competencies and contributions to the care of the frail older person. For tackling the multiple and complex needs of frail older persons, one specialist will never be sufficient. We need to be humbler, better organize clinical data according to the patient's values, and provide answers integrating multiple expertise. Novel approaches for more timely analysis of large datasets must be considered and implemented (11).

## Integration of Services Within the Community

During the very first months of the pandemic, the coronavirus spread quite easily within closed communities such as nursing homes. Persons living in this setting have been those who have probably paid the highest toll given (1) their intrinsic vulnerability and (2) the design and functioning of the infrastructures. For older adults with high level of complexity, typical geriatric syndromes were worsened by restriction policies and the interruption of activities considered as “non-essential” (e.g., physical therapy, occupational therapy, cognitive stimulation). A high incidence of COVID-19 was registered among professionals working in nursing homes, also because of inadequate procedural and organizational protocols and absence of qualified training. Many problems arose as a result of the hospital-centric model at the basis of most of the healthcare systems, negatively influencing the other knots of the network (e.g., primary care, long-term care) (12).

The sustainability of the hospitals itself has also been seriously stressed by the pandemic, sometimes leading them to the edge of collapsing. This situation resulted from public health interventions that have traditionally seen the hospital as the primary site of care. The hospital centralization in the network of care has implicitly been delegitimating, devaluing, and impoverishing the rest of the system, limiting the possibility of developing credible alternatives.

In this context, the difficulties encountered in the fight of COVID-19 during the first and second waves were not only evident at the hospital level. Social, organizational, and economic factors were also involved. Insufficient investments have been made in primary care services over the years. The example of the Region Lombardy (European epicenter of the first wave of COVID-19) is paradigmatic that, just a few years ago, developed a care model for the management of frail persons with chronic conditions centered on the hospital setting, initially without the proper (and needed) involvement of primary care physicians.

During the pandemic, specific guidelines for managing COVID-19 cases at home were not promptly available, leaving the primary care physicians isolated in front of the high number of potential cases. Furthermore, difficulties have been repeatedly reported in the access, planning, and organization of swab tests for people who got in contact with COVID-positive subjects. Even when special units designed for promoting continuity of care were set up, the lack of medical personnel made the service inefficient. In other words, during the pandemic, the existent hospital-centered model has enhanced the isolation of frail older persons in the community, leaving the often-unsupported primary care physicians facing the vast majority of cases.

Interestingly, geriatricians have been advocating for a different care model aimed at preventing the hospitalization of frail individuals through the strengthening of community services. To this extent, *ad hoc* units have been developed over the past years for supporting general practitioners in the assessment, diagnosis, and follow-up of most complex cases, for example, in France (13, 14), Japan (15), and United Kingdom (16). Furthermore, specific tools have been developed and disseminated over the past years for promoting the correct assessment of older individuals while raising awareness of the many neglected conditions of old age. A clear example of this attempt to bring the principles of geriatric medicine to the primary care setting is represented by the Rapid Comprehensive Assessment (17).

It is noteworthy that SARS-CoV-2 has also indirectly affected the health status of many older persons, even without infecting them. Frail older persons have multidimensional needs, ranging from the medical ones to others that are not usually considered in the clinical setting (i.e., social, psychological, affective, relational, cultural, spiritual, economical). Thus, even without having experienced the COVID-19 disease, frail older persons may have suffered the consequences of the physical distancing, social isolation, and disruption of continuity of care for their chronic conditions.

## Education and Training to Aging and Age-Related Conditions

As recently discussed by Searle and Rockwood (18), what is taught at the school of medicine is often very theoretical and far from real clinical life. There is still the tendency at describing and learning the “single disease” (in its epidemiology, clinical phenotype, diagnostic algorithm, therapeutic choices, etc.), but never the heterogeneous complexity of the today's clinical routine. It is essential also to fight the aforementioned ageism, to start looking at patients with a more holistic approach. This can only be done if the university and institutions in charge of the training of future generations of health professionals will substantially revise their programs and theoretical paradigms. In this period when scientific evidence is getting far and too speculative from the practical demands rising from the new generation of frail persons (19, 20), it might be crucial to privilege the presence of students in the clinical units instead of requiring the assimilation of sterile and often useless notions. To this extent, the development of specific curricula in geriatric medicine and grants to enhance teaching in geriatric medicine should be considered. Interprofessional education might also represent another way to learn geriatric medicine, facilitate the understanding of multidisciplinarity, and contrast ageism among healthcare professionals (21).

## Conclusions

The COVID-19 pandemic represents a stress test for healthcare systems worldwide. Geriatricians have been playing the Cassandra's role for many years, pointing at the criticalities of a system unable to correctly see the new demands coming from a diverse and growing population of patients. The crisis we have been living in should become an opportunity to transform the models of care by devoting adequate time and resources to the needs of frail older persons across the different settings. The attempt to change the current disease-based evaluation into a person-centered service has to be urgently made. Otherwise, the recent history and the dramatic number of deaths among frail older persons will have taught nothing.

## Data Availability Statement

The original contributions presented in the study are included in the article/supplementary material, further inquiries can be directed to the corresponding author.

## Author Contributions

All authors listed have made a substantial, direct and intellectual contribution to the work, and approved it for publication.

## Conflict of Interest

The authors declare that the research was conducted in the absence of any commercial or financial relationships that could be construed as a potential conflict of interest.
